# *Notes from the Field*: Counterfeit Percocet–Related Overdose Cluster — Georgia, June 2017

**DOI:** 10.15585/mmwr.mm6641a6

**Published:** 2017-10-20

**Authors:** Laura Edison, Amber Erickson, Sasha Smith, Gaylord Lopez, Stephanie Hon, Alexandra King, Nancy Nydam, J. Patrick O’Neal, Cherie Drenzek

**Affiliations:** ^1^Georgia Department of Public Health; ^2^Career Epidemiology Field Officer Program, CDC; ^3^North Central Health District, Georgia Department of Public Health, Macon, Georgia; ^4^Georgia Poison Center, Atlanta, Georgia.

On June 5, 2017, a Georgia North-Central Health District emergency department (ED) notified the Georgia Poison Center of six opioid overdoses and one death during the previous day. All patients had severe respiratory depression, loss of consciousness, or both, and some required high naloxone doses and mechanical ventilation. Two patients reported taking one or two pills that they believed to be Percocet, purchased without a prescription, on the street.

The Georgia Poison Center notified area hospitals and a Georgia Department of Public Health (GDPH) epidemiologist, who informed partners, including 1) health district epidemiologists, who worked with hospitals; 2) the Georgia Bureau of Investigation, which performed drug testing; 3) the High Intensity Drug Trafficking Area office, which notified law enforcement; 4) local coroners, who reported related deaths to GDPH; and 5) the GDPH Office of Emergency Medical Services (EMS), which notified EMS providers and the medical community. A coordinated communication effort led to two multiagency press conferences on June 6 to notify the public about the presence of the dangerous counterfeit pills.

A counterfeit Percocet cluster case was defined as 1) an opioid toxidrome (i.e., with central nervous system depression, respiratory depression, and pupillary miosis) requiring resuscitation, ventilation, naloxone, or all three; 2) a history of purchasing street pills; and 3) ingestion of as few as one or two pills, resulting in disproportionately severe central nervous system, respiratory, or cardiovascular depression occurring in a person evaluated by EMS or at an ED since June 1, 2017 (*1*). During June 6–13, EMS providers and EDs reported possible cases daily, and district epidemiologists reviewed medical records to determine whether patients met the case definition. Concomitant syndromic surveillance was conducted by reviewing Georgia statewide ED admission data received daily, using a text-search for drug overdose syndrome. This surveillance was used to determine whether the cluster extended beyond the initially identified area. Local law enforcement personnel delivered pills obtained from one patient to the Georgia Bureau of Investigation crime laboratory for chemical analysis.

Syndromic surveillance demonstrated a sharp increase in overdoses reported by EDs on June 5 (Figure). Chemical analysis of obtained pills identified cyclopropyl fentanyl and U-47700, two rare and potent illicit synthetic opioids. The source of the pill was not identified.

**FIGURE:**
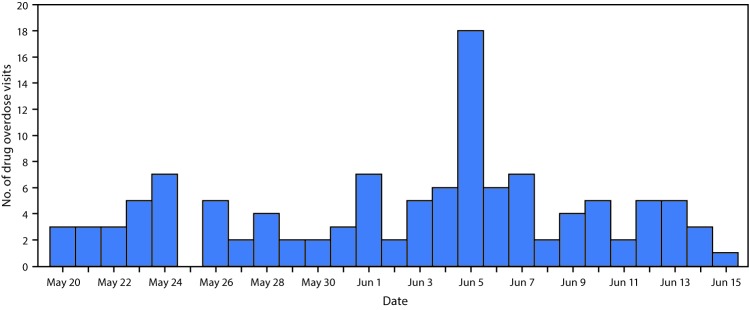
Drug overdose emergency department visits,* — North-Central Health District, Georgia, May–June, 2017 * The figure depicts all emergency department visits that met the overdose syndrome definition. Syndromic surveillance data cannot be used to determine whether these visits met the counterfeit Percocet cluster case definition, but they can monitor trends in overdoses. The North-Central Health District consists of 13 counties in central Georgia (http://northcentralhealthdistrict.org/).

Among the 37 possible cases reported initially (including five deaths), 27 cases (including one death) that occurred during June 4–13 met the counterfeit Percocet cluster case definition. Of the 27 patients, 16 (59%) were male, and 19 (70%) were black; median age was 34 years (range = 19–69 years). Symptoms included loss of consciousness (25 patients [93%]) and respiratory distress (22 [81%]). Twenty-five (93%) patients received naloxone, and 11 (41%) required intubation and mechanical ventilation. Routine urine drug screens were positive for multiple drugs in 16 (59%) patients; synthetic opioids are not detected by these screens.

E-mail descriptions of the pills and related overdoses were sent from the High Intensity Drug Trafficking Area office to law enforcement personnel and from GDPH to EMS and the medical community to alert all to the danger of these pills and how to prevent occupational exposure (*2*), to note that Georgia law specifies a naloxone standing order allowing anyone to purchase it (*3*), and to share the CDC opioid prescribing guideline with prescribers (*4*). Rapid identification, notification, public messaging, and a coordinated response among members of the health care community, public health agencies, and law enforcement personnel contributed to curtailing this outbreak.
